# Supplementary material for: Ultrafast carrier thermalization in lead iodide perovskite probed with two-dimensional electronic spectroscopy
Source: Nat Commun. 2017 Aug 29;8:376. doi: 10.1038/s41467-017-00546-z (PMC5575115; doi:10.1038/s41467-017-00546-z)
Supplement: Supplementary file 1 — Supplementary Information [file 41467_2017_546_MOESM1_ESM.pdf]

Description of Supplementary Files

File Name: Supplementary Information
Description: Supplementary Figures, Supplementary Notes and Supplementary References

File Name: Supplementary Movie 1
Description: $\mathbf{2DES\ data\ at\ 2 \cdot 10^{18} cm^{-3}.}$ Carrier relaxation in perovskites probed with 2DES. Each frame in the video represents a time delay t_2.

File Name: Supplementary Movie 2
Description: $\mathbf{2DES\ data\ at\ 2 \cdot 10^{19} cm^{-3}.}$ Carrier relaxation in perovskites probed with 2DES. Each frame in the video represents a time delay t_2.

File Name: Peer Review File

Supplementary Note 1: Estimate of carrier density

The carrier density n_0 after pulsed excitation is estimated using the absorption of the film a, the FWHM diameter of the Gaussian beam d, the laser power P, the laser repetition rate f, the average photon energy $E_{\mathrm{ph}} = \hbar\omega$ and the film thickness $t \approx 200$ nm. We estimate it according to

$$n_0 = \frac{a \cdot P}{E_{\mathrm{ph}} \cdot f} \cdot \frac{1}{\pi (d/2)^2 \cdot t} \tag{1}$$

Supplementary Note 2: Two-dimensional Electronic Spectroscopy

In transient absorption (TA) spectroscopy, also called pump-probe, the system interacts with two delayed pulses, the pump and the probe, and the nonlinear signal is measured as a function of detection (probe) frequency and pump-probe delay t_2.Two-dimensional electronic spectroscopy (2DES) is an extension of TA techniques which measures the nonlinear optical signal as a function of an extra dimension: the excitation (or pump) frequency. TA is a third-order nonlinear optical technique and as such detects four-wave mixing signals, which consist of three field-matter interactions followed by a nonlinear signal emission. In TA, the pump beam interacts with the sample twice and the non-collinear probe beam interacts with the sample once. The phase-matched nonlinear signal is emitted collinear with the probe beam, which acts as a phase-locked local oscillator (self-heterodyned configuration). By resolving the probe wavelength λ_{probe} with a spectrometer, one obtains a signal $S^{(3)}(t_2, \lambda_{\mathrm{probe}})$.

In 2DES a second pump pulse is introduced and the time delay between the two pump pulses (t_1) is scanned, thus resulting in a signal $S^{(3)}$ (t_1, t_2, λ_{probe}). During (t_1) the system is in a coherent superposition between ground and electronic excited states, and therefore the signal oscillates with approximately the optical carrier frequency as a function of t_1, corresponding to periods in the range of 1.3 to 2.5 fs in the visible range. Similarly to Fourier transform spectroscopy, by applying a Fourier transform over t_1 one obtains resolution over the excitation frequency. The nonlinear signal yields a correlation map between excitation (pump) and emission (probe) frequencies for a given waiting time t_2, which we report in wavelength: $S^{(3)}(\lambda_{\mathrm{pump}}, t_2, \lambda_{\mathrm{probe}})$. If the signal is heterodyne-detected, the complex-valued

third-order response function (convoluted with the excitation fields) can be recovered, therefore corresponding to the highest amount of information that can be extracted from a third-order technique. Indeed, the signal from TA and any other four-wave mixing technique can be obtained either as a cut or an integral of the 2DES signal.

2DES is conceptually analogous to multidimensional nuclear magnetic resonance (NMR) spectroscopy, which was developed in the 1970s and is a powerful tool in modern science. The main reason why its infrared and visible analogues flourished only in the early 2000s is that maintaining sufficient phase stability for the t_1 time period is substantially more challenging in these spectral ranges than it is in the radio frequency range used in NMR, due to the much shorter oscillation period of the electromagnetic waves, of the order of a few femtoseconds. Currently, a number of methods have been proven effective to overcome this challenge, employing different beam geometries and phase stabilisation strategies.

Our approach employs a set of birefringent wedges (Translating-Wedge-based Identical pulses eNcoding System – TWINS) to generate a pair of phase-locked collinear pulses with a controllable temporal delay between them, as detailed in reference (*1*). Briefly, the incoming pump beam has a polarization of 45 degrees with respect to the ordinary and extraordinary axes of the wedges (see Figure S1). The first plate of fixed thickness then introduces a fixed delay between the horizontal and vertical polarization components, ensuring that time zero will be accessible. Then, the beam propagates through the first pair of wedges, which is mounted with the optical axes rotated by 90 degrees with respect to the first fixed plate, so that temporal delay between vertical and horizontal components as a function of wedge insertion is in the opposite direction to that introduced with the fixed plate. Finally, the second pair of wedges is static and has the optical axis aligned such that no extra delay between the components is introduced, so that its function is to compensate for angular dispersion, front tilt and to optimize pulse compression. After the TWINS, a polarizer is used to select the 45 degree polarization. With this method we are able to generate a pair of collinear pump beams with remarkable phase stability, allowing us to perform 2DES using the pump-probe geometry. For characterization of the time zero and alignment of the TWINS, see reference (*1*).

By performing the experiment in the pump-probe geometry, the setup is also sensitive to the so-called pump perturbed free-induction decay when the pump beam arrives after the probe beam (*2*–*5*). In modern 2DES language, this signal originates from two third-order

double-sided Feynman diagrams which include one interaction with the probe followed by two interactions with the pump, and they correspond to non-rephasing pathways (free induction decay). Because there is a single interaction with the probe, the system is in a coherent superposition between ground and excited electronic states before the pump arrives. Consequently, the signal corresponds to an oscillation at the carrier frequency as a function of the time delay between pump and probe.

In our setup, the pump and probe beams are not phase locked, and as such it is not adequate to measure this signal, and if enough averages are taken the coherent oscillations eventually cancel out due to the phase fluctuations between pump and probe. The reason to acquire signal at negative delays is to reliably know where the pulse overlap region is, so that we can fully recover the signal we are interested in (i.e., that at positive times).

We stress that the inability to properly recover the "pump perturbed free-induction decay" is not a limitation of the setup, because the 2DES signal measured at positive times already contains the information from these pathways (*6*). Further, we note that the presence of this signal at negative delays cannot be a source of confusion because the coherent artefact clearly divides the negative from the positive regions of t_2, and when t_2 is positive causality ensures that the pathways contributing to the "pump perturbed free-induction decay" are identically zero.

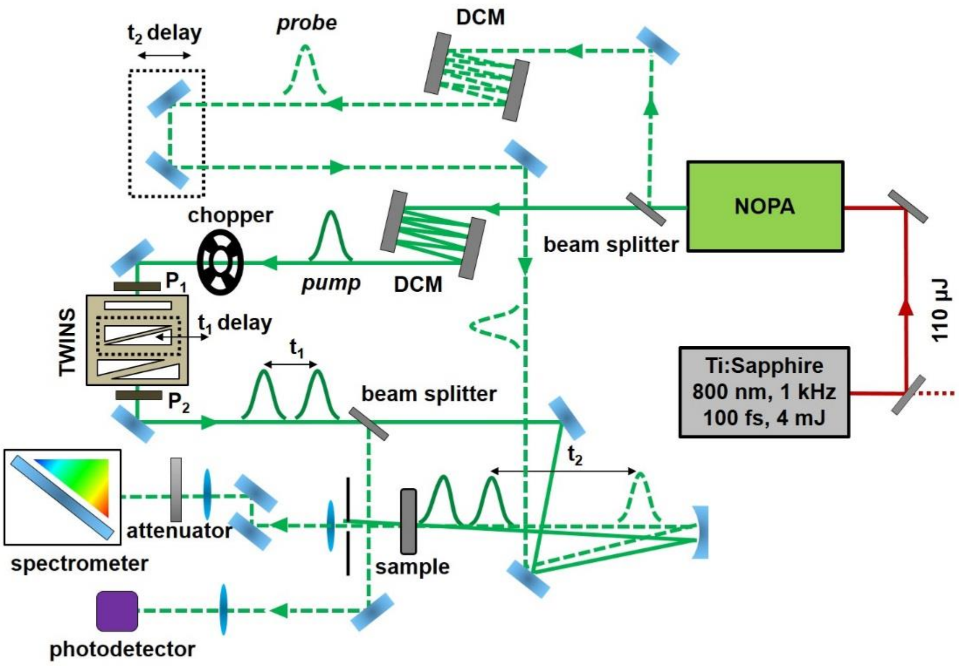

Supplementary Figure 1: 2DES setup layout. Set-up for femtosecond 2DES. NOPA: noncollinear optical parametric amplifier; DCM: double chirped mirror; t_1: linear translation for pump pulse pair delay; t_2: linear translation for pump-probe delay; P: polarizer; TWINS: Translating-Wedge-Based Identical Pulses eNcoding System.

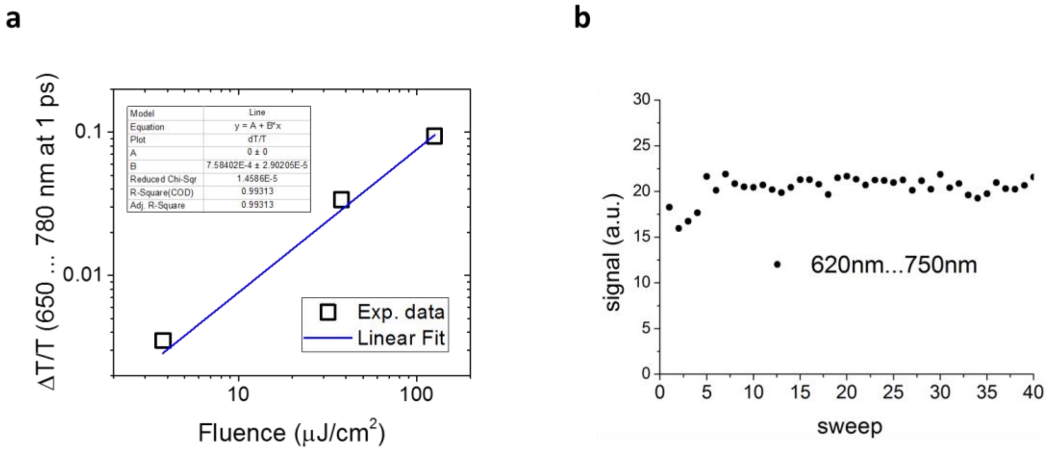

Supplementary Figure 2: Sample characterisation. (a) ΔT/T signal, integrated over the 650-780 nm wavelength range, at 1 ps time delay as a function of excitation fluence. The ΔT/T signal is proportional to the laser fluence indicating that we are measuring in the linear excitation regime. (b) 2DES signal at t_2 = 588 fs integrated over excitation and detection wavelengths plotted for different consecutive sweeps, where one sweep lasts for approximately 13 minutes. We observe no significant decrease of the signal, indicating that sample degradation is negligible over our measurement time interval.

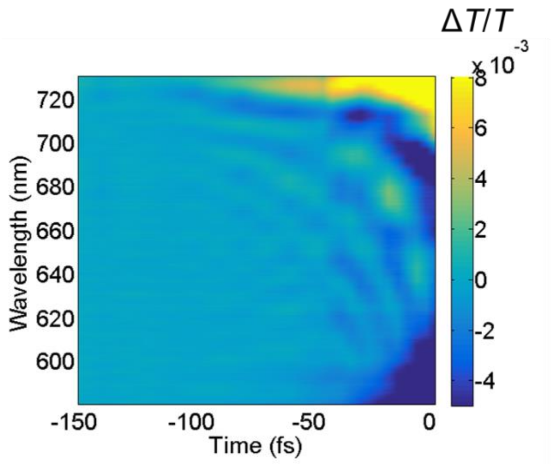

Supplementary Figure 3: Spectral oscillations in pump-probe experiment. ΔT/T map at negative time delays, i.e. with the probe pulse arriving before the pump pulse. We observe spectral oscillations with increasing frequency when approaching time zero, which are characteristic of pump-perturbed free induction decay.

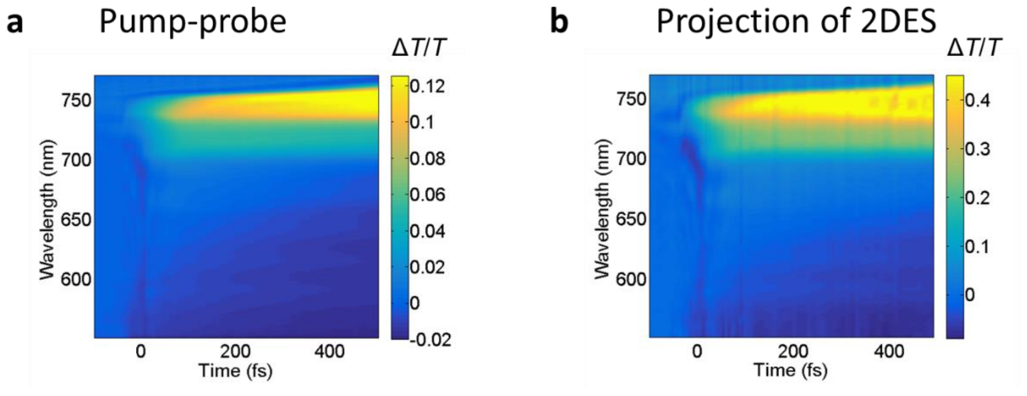

Supplementary Figure 4: Comparison of pump-probe and 2DES experiment. We calculate the projection of the 2DES data along the pump wavelength axis and plot it as a function of t_2 time and probe wavelength (**b**). We find a very good agreement with the pump-probe experiment (**a**).

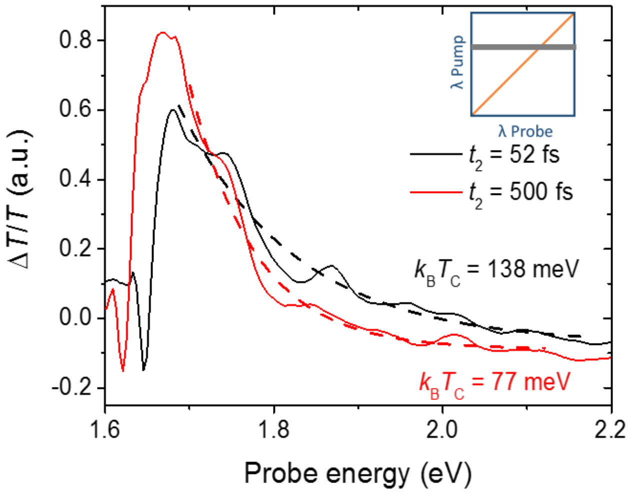

Supplementary Figure 5: Carrier temperatures at 625 nm pump wavelength. 2DES data at 625 nm pump wavelength for time delays t_2 of 52 fs (black continuous line) and 500 fs (red continuous line). The dashed lines represent exponential fits to the high energy tail of the spectra in order to extract carrier temperatures. We extract carrier temperatures of 1600 K for 52 fs and 890 K for 500 fs time delay.

Supplementary References

1. J. Réhault, M. Maiuri, A. Oriana, G. Cerullo, Two-dimensional electronic spectroscopy with birefringent wedges. *Rev. Sci. Instrum.* **85**, 123107 (2014).
2. C. H. Brito Cruz, J. P. Gordon, P. C. Becker, R. L. Fork, C. V. Shank, Dynamics of spectral hole burning. *IEEE J. Quantum Electron.* **24**, 261–269 (1988).
3. T. Kobayashi *et al.*, Real-Time Spectroscopy of Single-Walled Carbon Nanotubes for Negative Time Delays by Using a Few-Cycle Pulse Laser. *J. Phys. Chem. C.* **118**, 3285–3294 (2014).
4. S. Yan, M. T. Seidel, H.-S. Tan, Perturbed free induction decay in ultrafast mid-IR pump–probe spectroscopy. *Chem. Phys. Lett.* **517**, 36–40 (2011).
5. P. Hamm, Coherent effects in femtosecond infrared spectroscopy. *Chem. Phys.* **200**, 415–429 (1995).
6. J. D. Hybl, A. Albrecht Ferro, D. M. Jonas, Two-dimensional Fourier transform electronic spectroscopy. *J. Chem. Phys.* **115**, 6606–6622 (2001).
